# Impact of Hemodialysis on Sleep Disorders in Patients With End-Stage Renal Disease in a Tertiary Care Academic Hospital

**DOI:** 10.7759/cureus.44416

**Published:** 2023-08-30

**Authors:** Yogesh S Pawar, Vipul S Gattani, Kaustubh S Chaudhari, Bhavik Chheda, Ashok J Vankudre

**Affiliations:** 1 Department of Psychiatry, Dr. Vasantrao Pawar Medical College, Hospital and Research Centre, Nashik, IND; 2 Department of Neuropsychiatry and Sleep Medicine, SRP Neurosciences, Nashik, IND; 3 Department of Internal Medicine, Dr. Vasantrao Pawar Medical College, Hospital and Research Centre, Nashik, IND; 4 Department of Internal Medicine, Dr. Vaishampayan Memorial Government Medical College, Solapur, IND; 5 Department of Community Medicine, Dr. Vasantrao Pawar Medical College, Hospital and Research Centre, Nashik, IND

**Keywords:** conservative treatment, renal dialysis, kidney failure, insomnia, sleep

## Abstract

Introduction: Although hemodialysis (HD) has prolonged the survival of patients with end-stage renal disease (ESRD), it has also adversely affected the sleep and emotional state of these patients. We evaluated the impact of HD on sleep duration, quality, and other sleep-related disorders.

Methods: We recruited consecutive adult patients visiting our tertiary care dialysis unit. We included only ESRD patients who had an estimated glomerular filtration rate (eGFR) of <15 mL/min/1.73m^2^. We excluded patients with unrelated comorbidities or on medications that could affect sleep. Basic demographic information, anthropometric data, and appropriate lab investigations were obtained. Objective information related to their sleep duration and quality was asked using a predefined proforma. Subjective sleep scores were obtained by using the Pittsburgh sleep quality index (PSQI), Epworth sleepiness scale (ESS), and insomnia severity index (ISI). For comparison, the patients were divided into HD and conservative treatment (CT) groups based on their treatment modality. The baseline characteristics of the patients were noted. The Shapiro-Wilk test was used to test normality. Correlations were obtained by using Student’s t-test for parameters that were normally distributed and the Mann-Whitney-Wilcoxon test for those that were not.

Results: Of the 56 patients we studied, 59% were males. The average age and body mass index (BMI) were 45.7 years and 20.98 kg/m^2^, respectively. Overall, 41% of patients were assigned to the HD group, and the remaining to the CT group. The CT group had fewer comorbidities compared to the HD group. The average sleep duration was similar in both groups (HD: 6.64 hours, CT: 6.49 hours). There was a weak-to-moderate positive correlation between the sleep scores. Overall, one-half of the patients had excessive daytime sleepiness (EDS) (46.43%) and insomnia (48.21%), and two-thirds of them were poor sleepers (66.07%). Symptoms suggestive of sleep-disordered breathing (SDB) were seen in 25% of patients, restless legs syndrome (RLS) in 19.64% of patients, and periodic limb movement disorder (PLMD) in 44.64% of patients. Patients undergoing HD had poorer sleep quality compared to the CT group (p=0.038). The odds of developing poor sleep were 3.6 times higher in the HD group.

Conclusion: This cross-sectional study focuses on the quantification of objective and subjective deterioration of sleep quality in ESRD patients on HD. The prevalence of EDS (63.64%), insomnia (51.52%), and poor sleep quality (84.84%) in the HD group was more than the previously reported values. The PSQI, ESS, and ISI scores were higher in HD patients, indicating poorer sleep quality. Our study highlights the underestimation of sleep disorders in HD patients in underserved areas. The results warrant a meticulous evaluation of the same by a keen nephrologist, followed by referral to sleep providers where necessary.

## Introduction

Insomnia and sleepiness are global epidemics. The emergence of sleep disorders has traced the recent surge in the burden of non-communicable diseases (NCDs), such as diabetes mellitus (DM), hypertension (HTN), chronic kidney disease (CKD), and neurocognitive diseases [1]. Sleep disorders account for increased morbidity, frequent hospitalizations, reduced productivity at work, absenteeism, and accidents [2]. These direct and indirect costs add up to an already bloated budget of NCDs.

The causal relationship between sleep disorders and renal insufficiency is complex and often bidirectional. Sleep disturbances in CKD and end-stage renal disease (ESRD) are usually due to the stress of chronic disease, prolonged dialysis therapy, subclinical uremic encephalopathy (day-night reversal), disruption of sleep architecture, bone pain, and increased prevalence of sleep apnea [1,3-4]. Further, shorter sleep duration was significantly and independently associated with declining renal function [5]. This duality leads to a vicious cycle of progressive renal dysfunction and deteriorating sleep quality in patients with ESRD.

Hemodialysis (HD) has prolonged the overall survival of patients with ESRD. However, several sleep and emotional disturbances have been noted in patients on continued therapy [3,6]. According to a recent systematic review, the prevalence of poor sleep quality in CKD was 59% without replacement therapy and 67%-68% on dialysis [1]. The corresponding prevalence rates of insomnia were 48% and 46%-61%, respectively [1]. These rates were much higher than in the normal population and were reduced after renal transplant [1]. Sleep disorders are commoner in patients with ESRD, seen in almost 80% of cases [7-8]. A large multi-center study reported difficulty in initiating sleep (50%), maintaining sleep (59%), and waking up (49%) in 1,643 ESRD patients undergoing dialysis [9]. The inability to sleep has been ranked as a major concern for quality of life (QoL) by patients receiving renal replacement therapy [4,10].

Sleep disorders are generally ignored due to the subjective nature of symptoms, lack of awareness, and paucity of sleep clinics in developing countries. In patients with ESRD, sleep-related issues often tend to be sidelined amidst more obvious tasks such as dialysis.

There is a high degree of heterogeneity in the presentation of sleep disorders across ages, locations, and socioeconomic strata. This is reflected even in the existing studies exploring sleep issues in patients with CKD and ESRD [1]. This, compounded with inadequate training of general practitioners in diagnosing sleep disorders, leads to gross underdiagnosis and poor characterization of sleep disorders in ESRD.

Our study delves into a predominantly rural population with lower literacy and scarce access to specialized healthcare. Hence, our results reflect ESRD-related sleep disorders in an underexplored demographic. We evaluated the impact of HD on sleep quality in patients with ESRD. Additionally, we characterized the symptoms suggestive of insomnia, sleep-disordered breathing (SDB), restless legs syndrome (RLS), and periodic limb movement disorder (PLMD) in the same cohort.

## Materials and methods

This study was conducted in Dr. Vasantrao Pawar Medical College, Hospital and Research Centre, a tertiary care academic hospital in Nashik, India. The study was approved by the institutional ethics committee (IEC Reference No.: Dr. VPMCH&RC/IEC/80/2020-21). We screened consecutive adult patients (18-65 years) with ESRD who visited the nephrology unit for treatment. Consent was obtained from eligible patients.

We excluded patients previously diagnosed with sleep disorders or those with diseases or on medications that could alter sleep. Patients on HD and conservative treatment (CT) were included, and those who had a renal transplant were excluded. Patients with epilepsy, recently diagnosed (within six months) cardiovascular and cerebrovascular disease, liver diseases, cognitive and psychiatric disorders, and with an acute history of pain or intercurrent illnesses were excluded from the study.

If eligible, the initial workup was conducted by the Nephrology Unit of the Medicine Department and then referred to the Neuropsychiatry Division for evaluation of sleep disorders. A predefined proforma was used to record the patient’s history. We recorded the age, gender, education, occupation, marital status, and family type of the patients. Smoking, tobacco chewing, and alcohol habits were noted. The history of CKD and any concurrent NCDs was noted both for the patient and their family. The patient's vital, anthropometric measurements, and neck circumference were measured.

We considered patients who had ESRD according to the Kidney Disease Improving Global Outcomes classification. Accordingly, only those patients who had an estimated glomerular filtration rate (eGFR) <15 mL/min/1.73m^2^ were included. Patients receiving dialysis for more than three months were assigned to the HD group, while the remaining patients were assigned to the CT group. All patients received in-center dialysis in a daycare facility. An average dialysis duration was four hours and took place between 7 AM and 7 PM. Patients received dialysis two to three times per week.

We obtained the patient’s detailed sleep history, including sleeping habits, duration, and quality. The nature of their sleep complaints too was documented. Additionally, sleep was subjectively scored using three validated questionnaires, viz., Pittsburgh sleep quality index (PSQI), Epworth sleepiness scale (ESS), and insomnia severity index (ISI). These questionnaires were explained to the patients and assistance was provided while they responded to the questions. If needed, the questions were explained to the patients in ways or situations that they could relate to based on their sociocultural status. The answers were either self-reported or obtained by interviewing.

The PSQI is a seven-question questionnaire evaluating the patient's sleep quality reported over the past month. The components tested include sleep duration, habitual sleep efficiency, sleep latency, subjective sleep quality, sleep disturbances, daytime dysfunction, and sleep medication. PSQI provides a global score on a scale of 0-21, with lower scores suggesting better sleep quality. People with scores less than five are "good sleepers" and those above are "poor sleepers" [11].

The ESS is a self-reported questionnaire that identifies a person's chances of dozing off or falling asleep in general and in eight different day-to-day scenarios. A total score of 0-24 is obtained by adding the eight individual situational sleep propensity scores. A score of 10 or more suggests a stronger likelihood of daytime sleepiness and is of concern [12].

The ISI has seven questions that inquire about a person’s sleep experience over the past two weeks. The tested areas include sleep (onset, maintenance, and awakening) issues, interference with daytime functioning, sleep issues noticed by others, overall sleep dissatisfaction, and associated distress. Scores of 0-7, 8-14, 15-21, and 22-28 indicate varying levels of insomnia, namely, not significant, subthreshold, moderate, and severe insomnia, respectively [13].

Additionally, patients were asked questions about symptoms suggestive of SDB, RLS, and PLMD. We also asked for symptoms related to sleep apnea, bruxism, dream enactment, etc. If diagnosed with sleep difficulty, patients were offered treatment as deemed clinically necessary.

We presented baseline characteristics as absolute values and percent proportions, mean and standard deviation (SD), or median and interquartile range (IQR). We used the Shapiro-Wilk test for normality and found that ESS followed a normal distribution, while PSQI and ISI followed non-normal distribution. Hence, parametric tests were used for ESS, and non-parametric tests were used for PSQI and ISI. Similarly, correlations involving ESS were done using the Pearson correlation coefficient, and those for PSQI and ISI were done using Spearman’s rank correlation coefficient.

Unpaired Student’s t-test was used for basic demographics such as age, body mass index (BMI), and sleep duration, and the variables were reported as mean±SD. Normally distributed variables were tested using Student’s t-test and presented as mean±SD. Non-normally distributed variables were tested using the Mann-Whitney-Wilcoxon test and presented as median and IQR. The odds ratios (OR) for different sleep scores between the HD and CT groups were calculated.

Statistical Package for the Social Sciences (SPSS, IBM SPSS Statistics for Windows, Armonk, NY) and Microsoft Excel software for Windows OS were used for data analysis. Statistical significance was considered when p value was less than 0.05.

## Results

Of the 56 patients included in the study, 59% were males; 65% of them in HD, and 55% in CT groups. The average age across both groups was 45.7±13.8 years (Table 1). There was a significant difference in the mean ages of the HD and CT groups (mean difference=15.69 years, p<0.001). The average BMI of the patients was 20.98±2.27 kg/m^2^. Overall, 71.43% of patients were married, and 76.79% of patients were either illiterate or were educated up to primary school. Of the 56 patients, 23 (41.07%) patients were on HD. Comorbidities such as DM and HTN were present in 30 (53.56%) patients. The CT group had fewer comorbidities compared to the HD group (39.13% versus 63.63%).

**Table 1:** Basic demographic characteristics The sleep quality, excessive daytime sleepiness, and insomnia values were derived from PSQI, ESS, and ISI scores, respectively. Abbreviations: BMI: body mass index, CT: conservative treatment, DM: diabetes mellitus, ESRD: end-stage renal disease, ESS: Epworth sleepiness scale, HD: hemodialysis, HTN: hypertension, IQR: interquartile range, ISI: Insomnia severity index, PLMD: periodic limb movement disorder, PSQI: Pittsburgh sleep quality index, RLS: restless legs syndrome, SD: standard deviation, SDB: sleep-disordered breathing

		HD Group	CT Group
		n=23 (41.07%)	n=33 (58.93%)
Age (in years) (Mean±SD)		52.12±11.2	36.43±11.9
Gender (no, %)	Male	15 (65.22)	18 (54.55)
	Female	8 (34.78)	15 (45.45)
BMI (kg/cm^2^) (Mean±SD)		20.45±2.6	21.74±1.4
Marital Status (no, %)	Single	6 (18.18)	6 (26.09)
	Married	24 (72.73)	16 (69.57)
	Divorced	3 (9.09)	1 (4.35)
Education (no, %)	Illiterate	8 (24.24)	2 (8.7)
	Primary	17 (51.52)	16 (69.57)
	Secondary	3 (9.09)	2 (8.7)
	Graduate	3 (9.09)	3 (13.04)
	Post Graduate	2 (6.06)	0 (0)
Comorbidities (no, %)	ESRD	12 (36.36)	14 (60.87)
	ESRD with DM	3 (9.09)	3 (13.04)
	ESRD with HTN	9 (27.27)	2 (8.7)
	ESRD with DM with HTN	9 (27.27)	4 (17.39)
Sleep duration (Mean±SD)		6.64±0.6	6.49±0.7
Sleep scores	PSQI (Median, IQR)	9 (5-13)	6 (4-9)
	ESS (Mean±SD)	10.67±3.81	8.87±3.58
	ISI (Median, IQR)	8 (6-14)	6 (4-11)
Sleep quality (n, %)	Good sleepers	5 (15.15)	9 (39.13)
	Poor sleepers	28 (84.84)	14 (60.87)
Excessive daytime sleepiness (n, %)	Present	21 (63.64)	9 (39.13)
	Absent	12 (36.36)	14 (60.87)
Insomnia (n, %)	Present	17 (51.52)	10 (43.48)
	Absent	16 (48.84)	13 (56.52)
Symptoms suggestive of (no, %)	SDB	7 (30.44)	7 (21.21)
	RLS	7 (30.44)	4 (12.12)
	PLMD	11 (47.83)	14 (42.42)

The mean sleep duration was 6.64±0.6 hours in the HD group and 6.49±0.7 hours in the CT group. The average overall sleep scores recorded were: PSQI of 8.35 (SD=4.33), ESS of 9.92 (SD=3.79), and ISI of 8.93 (SD=4.9). There was a weak to moderate positive correlation between the overall sleep scores: PSQI and ESS (rho=0.33, p=0.012), PSQI and ISI (rho=0.287, p=0.031), and ESS and ISI (rho=0.20, p=0.131).

Nearly half of all ESRD patients had excessive daytime sleepiness (EDS) (46.43%) and insomnia (48.21%), based on ESS and ISI scores, respectively. Two-thirds of patients were poor sleepers (66.07%), based on the PSQI score. Symptoms suggestive of SDB were seen in 25%, RLS in 19.64%, and PLMD in 44.64% of patients.

The sleep scores varied inversely with eGFR across both HD and CT groups (Table 2). There was a strong negative correlation between eGFR and PSQI of the CT group (rho=-0.77, p<0.001) and a moderate negative correlation between eGFR and ESS of the HD group (r=-0.54, p=0.001).

**Table 2:** Linear correlation between sleep scores and eGFR of HD and CT groups Abbreviations: CT: conservative treatment, eGFR: estimated glomerular filtration rate, ESS: Epworth sleepiness scale, HD: hemodialysis, ISI: insomnia severity index, PSQI: Pittsburgh sleep quality index

		PSQI		ESS		ISI	
		Spearman's rho	p-value	Pearson's r	p-value	Spearman's rho	p-value
eGFR	HD Group	-0.41	0.017	-0.54	0.001	-0.36	0.037
	CT Group	-0.77	<0.001	-0.25	0.245	-0.26	0.233

The patients in the HD group were more significantly poor sleepers when compared with the CT group (p=0.038) (Table 3). There was a weak to moderate inverse correlation between eGFR and ESS and ISI scores in both groups. Further, although the severity of insomnia and daytime sleepiness was more in patients of HD than those of the CT group, such differences were not significant (p=0.075 and p=0.078) (Table 3). We noted much higher odds of developing poor sleep (OR=3.6), daytime sleepiness (OR=1.83), and insomnia (OR= 1.38) in the HD group (Table 4).

**Table 3:** Comparison between sleep scores of the HD and CT groups Abbreviations: a: Mann-Whitney-Wilcoxon test, b: Student's t-test, CT: conservative treatment, ESS: Epworth sleepiness scale, HD: hemodialysis, IQR: interquartile range, ISI: insomnia severity index, PSQI: Pittsburgh sleep quality index, SD: standard deviation

	PSQI^a^ (Median, IQR)	ESS^b^ (Mean±SD)	ISI^a^ (Median, IQR)
HD Group	9 (5-13)	10.67±3.81	8 (6-14)
CT Group	6 (4-9)	8.87±3.58	6 (4-11)
p-value	0.038	0.078	0.075

**Table 4:** Odds of developing poorer sleep scores in the HD group compared with the CT group. Abbreviations: CI: confidence interval, CT: conservative treatment, ESS: Epworth sleepiness scale, HD: hemodialysis, ISI: insomnia severity index, OR: odds ratio, PSQI: Pittsburgh sleep quality index

Score	OR	95% CI	p-value
PSQI	3.6	1.013-12.788	0.048
ESS	1.83	0.615-5.466	0.277
ISI	1.38	0.474-4.029	0.554

## Discussion

Through this cross-sectional study, we attempted to address the scarcity of data on sleep disorders in patients with ESRD from low-resource settings. We compared the subjective sleep scores and associated sleep disorders in patients on HD and those receiving CT. Our study population was predominantly rural, had lower literacy, and belonged to the lower socio-economic strata. They had limited access to healthcare and almost no access to specialized sleep care. While a few studies have addressed sleep issues in a similar demographic [3,14], the data on the comparative impact of HD is scarce.

Since the biochemistry of patients with renal transplants is less deranged than those with renal failure, including the former could lead to underprediction of the sleep burden. Hence, patients who had renal transplants were excluded. The higher age in our HD group could be due to a higher incidence of comorbidities and progression of the disease [15-16]. Consequently, the CT group was younger and had a lower incidence of comorbidities. The average sleep durations were almost similar in both groups. Dialysis shifts can adversely influence the sleep profile of patients in the HD group. However, evidence in that respect has been equivocal [3,8].

The EDS identified by ESS had better reliability and sensitivity than objective sleep measures [12]. Pathological sleepiness (ESS≥17) [12] was seen in four patients in the HD group and was absent in the CT group. Like the ESS, PSQI too had higher sensitivity (89%) and specificity (86.5%) in the identification of poor sleepers than objective sleep measures [17].

In our study, all three sleep scores showed a weak-to-moderate positive correlation between themselves and showed a negative correlation with eGFR values. Further, the scores were higher in the HD group when compared with the CT group, although only PSQI scores were significantly higher. This indicates that sleep quality, daytime sleepiness, and insomnia were severe in patients on HD.

In the HD group, EDS was seen in 63.64%, insomnia in 51.52%, and poor sleep quality in 84.84% which were much higher than previously reported values of 12.8%, 19.5%, and 68.2% reported by previous workers from India [14]. The prevalence of insomnia was also higher than the 45% reported in Italy [18]. All these proportions were higher in the HD group when compared with the CT group.

There were 3.6-fold higher odds of developing poor sleep quality when a patient was on HD. The odds of developing EDS and insomnia were also high with HD, but unlike poor sleep quality, they were not significant. This is like the correlation of sleep scores and treatment method, where only the PSQI difference between the two groups was significant.

Symptoms suggestive of RLS, PLMD, and SDB were slightly higher in the HD. This was similar to the findings of previous workers [19-21]. An increase in the number of disability days, waking up for dialysis shifts, the stress of chronic disease, and impaired overall QoL have been additionally implicated in impaired sleep [22]. QoL impairment has been linked to lower socio-economic status and the presence of comorbidities [23]. Chronic bone pain in dialysis patients too has been associated with impaired mobility and sleep disturbances [24-25]. CKD is known to blunt the phycological rise in serum melatonin [26]. Previously, lower melatonin levels have been reported in CKD and ESRD [27]. These values did not improve with HD or renal transplant [28]. This results in impaired circadian rhythm and could serve as an explanation for poor sleep quality in HD patients. While HD impairs sleep, reduction in sleep duration and quality have been associated with progressive eGFR decline, which further impairs sleep quality [29-30], as seen in our study.

Our study provides vital information about the prevalence of sleep disorders in dialysis patients from underserved areas. However, it does have limitations. Since this is a single-center study, our conclusions cannot be generalized before larger multi-center studies are conducted. Further, the cross-sectional design limits our understanding of how sleep deterioration progresses with ongoing hemodialysis. While the average age of patients on HD is higher than those in the CT group, a study with age-matched groups would provide a better overview of the independent influence of HD on sleep quality. We took efforts to make the questions and scenarios mentioned in the questionnaires more relatable to the illiterate patients. However, appropriately modified questionnaires can be developed to address such challenges in the future. Further, in our tertiary center, most patients are referred from peripheral centers for specialist opinion for refractory disease, for complications, or for receiving dialysis. Hence, it is likely that sleep-related disorders are more prevalent and severe in the selected cohort. However, this could present a scenario faced by most tertiary dialysis centers serving underserved areas. Polysomnography and cerebral imaging could certainly add value to sleep assessment. However, subjective assessment using questionnaires suffices our current aim of evaluating sleep quality in patients on HD and is also inexpensive. Future studies can delve further into sleep disorders, by using polysomnography, cerebral imaging, and questionnaires for specific sleep disorders.

## Conclusions

Our study corroborates the findings of most of the previous studies. We found a higher proportion of sleep disturbances and poorer sleep quality in our HD patients when compared with the CT patients. This could reflect the poor detection of sleep disorders in patients with renal failure especially those from underserved areas. Larger, multi-centric, prospective studies are essential to validate this data. A keen nephrologist would benefit from looking out for subtle sleep disturbances in patients on HD. Considering the bidirectional influence of renal failure and sleep, an earlier referral, diagnosis, and treatment of sleep disorders could impede the progress of renal failure and improve their patients’ QoL.
